# Sex-Dependent Effects of the Intake of NOVA Classified Ultra-Processed Foods on Syndrome Metabolic Components in Brazilian Adults

**DOI:** 10.3390/nu14153126

**Published:** 2022-07-29

**Authors:** Elma Izze da Silva Magalhães, Bianca Rodrigues de Oliveira, Lívia Carolina Sobrinho Rudakoff, Vitória Abreu de Carvalho, Poliana Cristina de Almeida Fonseca Viola, Soraia Pinheiro Machado Arruda, Carolina Abreu de Carvalho, Carla Cristine Nascimento da Silva Coelho, Maylla Luanna Barbosa Martins Bragança, Heloisa Bettiol, Marco Antônio Barbieri, Viviane Cunha Cardoso, Alcione Miranda dos Santos, Renata Bertazzi Levy, Antônio Augusto Moura da Silva

**Affiliations:** 1 Postgraduate Programme in Collective Health, Federal University of Maranhão, São Luís 65020-070, Maranhão, Brazil; oliveirarodrigues00@gmail.com (B.R.d.O.); livia.rudakoff@ifma.edu.br (L.C.S.R.); carv.vitoria@gmail.com (V.A.d.C.); polianafonseca@ufpi.edu.br (P.C.d.A.F.V.); carolina.carvalho@ufma.br (C.A.d.C.); carlacristinecoelho@gmail.com (C.C.N.d.S.C.); mayllabmartins@gmail.com (M.L.B.M.B.); alcione.miranda@ufma.br (A.M.d.S.); aamouradasilva@gmail.com (A.A.M.d.S.); 2 Department of Nutrition, Federal University of Piauí, Teresina 64049-550, Piauí, Brazil; 3 Postgraduate Programme in Nutrition and Health and Postgraduate Programme in Collective Health, Estadual University of Ceará, Fortaleza 60714-903, Ceará, Brazil; soraia.arruda@uece.br; 4 Postgraduate Programme in Child and Adolescent Health, University of São Paulo, Ribeirão Preto 14048-900, São Paulo, Brazil; hbettiol@fmrp.usp.br (H.B.); mabarbieri@fmrp.usp.br (M.A.B.); vicuca@fmrp.usp.br (V.C.C.); 5 Department of Preventive Medicine, Medicine School, University of São Paulo, São Paulo 04023-062, São Paulo, Brazil; rlevy@usp.br

**Keywords:** ultra-processed foods, metabolic syndrome, abdominal obesity, low HDL-cholesterol, longitudinal studies

## Abstract

Longitudinal studies evaluating the relationship between UPF consumption and the incidence of Metabolic Syndrome (MetS) and its components are still scarce. This study aimed to evaluate the effect of UPF consumption on the incidence of MetS and its components in adults. A prospective study was conducted with 896 participants from the 1978/79 Ribeirão Preto cohort, São Paulo, Brazil. UPF consumption was evaluated in %kcal and %g at ages 23–25 years. Incidence of MetS and its components were estimated at ages 37–39 years, according to the Joint Interim Statement criteria. Poisson regression was used to assess associations, and interactions with sex were investigated. UPF consumption had no association with MetS (%kcal Adjusted PR: 1.00; 95% CI: 0.99–1.01; %g Adjusted PR: 1.00; 95% CI: 0.99–1.01). However, women with higher UPF consumption, in %kcal and %g, had a higher risk of abdominal obesity (%kcal: *p* = 0.030; %g: *p* = 0.003); and women with higher UPF consumption, in %g, had a higher risk of low HDL-cholesterol (*p* = 0.041). For the other components of MetS, no significant associations were observed in either sex. These findings suggest evidence of no association between UPF consumption and MetS; however, consumption of UPF was associated with increased WC and low HDL-c, but only in women.

## 1. Introduction

According to the definition of the NOVA system of food classification, ultra-processed foods (UPF) are formulations of ingredients, mainly of exclusive industrial use, resulting from a series of industrial techniques and processes [1]. UPF consumption has increased worldwide, which represents a public health problem for several reasons [2,3]. In general, UPFs have higher energy density, higher sugar, sodium, saturated and trans fats and low amount of proteins, fibers, and micronutrients. Moreover, they have additives (dyes, thickeners, sensory enhancers, among others), which are never or rarely used in the kitchen, whose purpose is to heighten the product attractiveness, palatability, and shelf life [1].

Parallel to the increase in UPF consumption, the prevalence of metabolic syndrome (MetS) increased in recent decades, also becoming an important public health problem [4,5]. MetS is a cluster of metabolic risk factors, including abdominal obesity, hyperglycemia, hypertriglyceridemia, hypertension, and low levels of high-density lipoprotein cholesterol (HDL-c). This condition is associated with increased risk of cardiovascular disease, type 2 diabetes, and mortality [5]. In this sense, lifestyle interventions, which include healthy eating, physical activity, non-smoking, and body weight control, have been pointed out as a strategy for MetS prevention [6].

Evidence has shown that UPF consumption contributes significantly to adverse health effects, such as the increase in chronic non-communicable diseases (NCDs) and all-cause mortality [7,8]. In this sense, cross-sectional studies reported an association between UPF consumption and MetS [9,10,11]. However, to date, evidence on the role of UPF consumption in the incidence of MetS and its components is scarce [12], reinforcing the need to better investigate this relationship.

In view of the increasing UPF consumption and its potential negative health impacts, this study aimed to evaluate the effect of UPF consumption on the incidence of MetS and its components in adults in a Brazilian cohort.

## 2. Materials and Methods

### 2.1. Study Design and Sample

This was a prospective study with data from a birth cohort initiated in 1978/79 in the city of Ribeirão Preto, state of São Paulo, Southeast region of Brazil. Cohort data were used for birth (baseline), follow-up at 23–25 years (fourth phase) and follow-up at 37–39 years of age (fifth phase). At birth, 6973 births were evaluated in hospitals of mothers living in Ribeirão Preto. In the fourth phase of the cohort, a sample of one in three individuals was evaluated between 2002 and 2004, representing 31.8% of the original cohort. In the fifth phase, whose data collection occurred between 2016 and 2017, 1775 participants were evaluated. Details on the eligible individuals who were recruited and evaluated at each stage of this cohort are available in other publications [13,14].

In the 1978/79 Ribeirão Preto cohort, data of single births, evaluated in the fourth and fifth phase of the study, with collected information on food consumption in the fourth phase and on components of MetS in the fourth and fifth phase were available for 1025 participants. Of these 1025 participants, individuals diagnosed with MetS (*n* = 123) and implausible data on food consumption (±3 standard deviation of the mean daily energy intake: *n* = 6) at follow-up at 23–25 years were excluded, resulting in a sample of 896 participants in the main analyses. Subsequently, of the 896 participants considered in the main analyses, we excluded individuals diagnosed with each of these components at follow-up at 23–25 years (Increased waist circumference = 221; increased triglycerides = 43; low high density lipoprotein cholesterol = 331; increased blood pressure = 122; increased glucose = 21) to estimate the incidences of each MetS component.

### 2.2. Exposure Variable

The UPF consumption at the follow-up at 23–25 years, as the exposure variable, was continuously evaluated considering the percentage of energy (%kcal) and grams (%g) from the UPFs consumed by the participants in the daily total of calories and grams, respectively.

Food consumption data were obtained by distributing a semi-quantitative Food Frequency Questionnaire (FFQ) regarding food consumption in the last 12 months. This FFQ was not validated, but was adapted for use in non-communicable chronic disease prevention programs aimed at adults, including the age group evaluated in this study [15].

The FFQ consisted of 83 food items (Supplementary Board S1) and their intake number of times (0 to 9 times) and frequency of consumption (daily, weekly, or monthly) and the size of the mean reference portion. Thus, participants were asked to report the number of times per day, week, or month in which each item was consumed and whether the portion usually consumed was small (less than the mean reference portion), mean (equal to the mean portion of reference), or large (greater than the mean portion of reference). From the dietary portions, the percentage distribution of the weights equivalent to the homemade measures mentioned in the 24 h dietary recall were determined and applied during the FFQ preparation stage. The mean portion presented for each food item represented the 50th percentile and the small and large portions the 25th and 75th percentiles, respectively [16]. The FFQ was applied by nutritionists, using a photo album to [17] help estimate the portions consumed by the participants.

To estimate food consumption in grams (g) per day, the consumption frequencies of each daily food item were obtained (with the appropriate conversions, whether based on weekly or monthly consumption) and multiplied by the size of the portion. From the values in grams, the intake of nutrients and energy of each food was estimated by using food composition tables [18,19,20]. Regarding alcoholic beverages, energy from alcohol (7 kcal/g) was also computed in estimating the energy value of these food items.

Foods were grouped according to the NOVA classification as follows: in natura or minimally processed foods, processed culinary ingredients, and processed foods and ultra-processed foods. The group of in natura or minimally processed foods and processed culinary ingredients were considered jointly, being termed as “culinary preparations” [21].

It is important to mention that since at the time of research planning the NOVA classification had not yet been published, the FFQ used in the study was not designed to classify food according to the degree of processing and consequently to accurately measure UPF intake. Thus, some considerations regarding the consumption estimates of some food items needed to be taken into account. When foods from different groups were grouped in the FFQ, the contribution of these foods was divided into more than one group, using as a parameter the consumption of said foods in the State of São Paulo, according to the estimates observed in the Brazilian Family Budget Survey (2002–2003) [22], which was conducted relatively close in time to the period of data collection in our study. For example, in the case of the food item “oatmeal and granola”, 12% was allocated for the group of in natura or minimally processed foods (concerning to oatmeal) and 88% was allocated for the group of UPF (concerning granola).

Finally, the percentage contribution in grams (%g) and energy (%kcal) of each food group was estimated for the total weight and total calories of the diet, respectively.

### 2.3. Outcome Variable

The diagnosis of MetS and its components, in the study outcomes, was determined in the follow-ups at 23–25 years (baseline) and 37–39 years.

Waist circumference (WC) was measured in centimeters using an inelastic fiberglass tape. To obtain WC measurement, the smallest perimeter between the ribs and the iliac crest was considered while the participant was standing with a relaxed abdomen at the end of a normal expiration. In the absence of natural waist, the measurement was performed at the level of the umbilical scar.

To determine the biochemical components of MetS, in the 23–25 years follow-up, 40 mL of blood was collected after 12 h of fasting of the participant. In the 37–39 years follow-up, the same collection was performed in a non-fasting state. Blood glucose was measured by the human diagnostic calorimetric enzymatic method GOD/PAP with a coefficient of variation of 4.2%. High-density lipoprotein cholesterol (HDL-c) and triglycerides were determined by the same method using the Dade Behring XP device and Dade Behring Dimension reagents.

To gauge blood pressure, the Omron^®^ digital sphygmomanometer model 740 was used. Three checks were performed with 15 min intervals between each, and the individuals remained seated with their left arm at heart height. The mean between the last two measurements was considered to represent the blood pressure of the participants.

In the 23–25 year-old follow-up, the criterion for diagnosing MetS was considered to be a change in at least three components according to parameters established by the Joint Interim Statement (JIS) [23] which are as follows: WC ≥ 90 cm for men and ≥80 cm for women; triglycerides ≥ 150 mL/dL or use of antilipemic medicine; HDL-c < 40 mg/dL for men and <50 mg/dL for women or use of antilipemic medication; systolic ≥ 130 mmHg or diastolic arterial pressure ≥ 85 mmHg or use of antihypertensive medication; and fasting blood sugar ≥ 100 mg/dL or use of antihyperglycemic medication. The use of antilipemic and antihyperglycemic drugs was obtained by self-report with the application of a questionnaire to the participants. In the 37–39 years-old follow-up, due to the non-fasting requirement during blood sample collection, the parameters established by the JIS [23] were considered, except for the evaluation of triglycerides for which it was considered as altered if ≥175 mL/dL [24,25].

### 2.4. Birth Variables

Data on pregnancy, delivery, and newborn, as well as demographic, socioeconomic, and maternal data, were obtained with a standardized questionnaire applied shortly after delivery by trained personnel at the time of birth in 1978/1979. In this study, the following variables were considered: maternal smoking during pregnancy (yes/no); number of prenatal consultations (0 consultations/1 to 5 consultations/≥6 consultations); gestational age (<37 weeks/≥37 weeks); type of delivery (cesarean/vaginal); birth weight (<2500 g/≥2500 g); length at birth (<50 cm/≥50 cm); sex (male/female); family income (in quartiles); parity (1 delivery/2 to 4 deliveries/≥5 deliveries); maternal age (<20 years/20 to 34 years/>35 years); maternal education (0 to 4 years of schooling/5 to 8 years of schooling/9 to 11 years of schooling/≥12 years of study); maternal marital status (with partner/without partner); maternal occupation (not manual/qualified manual/unqualified manual).

### 2.5. Socioeconomic, Demographic, and Lifestyle Variables at 23–25 Years

At the age of 23–25 years, demographic, socioeconomic, and lifestyle data were obtained by trained personnel through structured questionnaires. For this study, the following variables and respective categories were considered: sex (male/female); self-reported skin color (white/black/mixed race/Asian or indigenous); age (23 years/24 years/25 years); schooling (0 to 8 years of study/9 to 11 years of study/≥12 years of study) marital status (with partner/without partner); family income (<5 minimum wages/5 to 9.9 minimum wages/≥10 minimum wages); alcohol consumption (yes/no); smoking (yes/no); and level of physical activity (high/moderate/low). The level of physical activity was evaluated and defined according to the guidelines of the International Physical Activity Questionnaire (IPAQ) [26].

### 2.6. Data Analysis

A statistical analysis was performed using the software Stata 14.0^®^ (Stata Corporation, College Station, TX, USA). To minimize bias due to follow-up losses, weighting was performed by the inverse of the selection probability. For the weighting, only variables collected at birth were considered (maternal smoking during pregnancy, number of prenatal visits, gestational age, type of delivery, birth weight, birth length, sex, family income, parity, maternal age, maternal education, maternal marital status, and maternal occupation), as previously described.

The continuous variables were described as measures of central tendency and dispersion (means and standard deviations) and categorical variables by absolute and relative frequencies.

The mean consumption of UPF in %g and %kcal at 23–25 years was compared according to socioeconomic, demographic, and lifestyle characteristics also at 23–25 years, by using the Student’s *t* and ANOVA tests. The frequencies of metabolic syndrome and each of its components at 37–39 years were also compared according to socioeconomic, demographic, and lifestyle characteristics at 23–25 years using Pearson’s chi-square or Fisher’s exact tests. For the statistical tests, a significance level of 5% was considered.

To evaluate the effect of UPF intake on the incidence of MetS and its components, Poisson regression models were adjusted with robust variance, with the variables MetS and each of its components used as an outcome. In each model, the crude and adjusted risk ratios and their respective 95% confidence intervals were estimated.

For confounder control, the independent variables included in the adjusted model were identified by constructing the Directed Acyclic Graph (DAG) (Supplementary Figure S1) in the Daggity version 3.0 program. Based on the back door criterion [27], we identified the need for minimum adjustment for sex, skin color, age, education, marital status, family income, alcohol consumption, smoking, and level of physical activity. In the analyses with the consumption of UPF in %g, an additional adjustment was made for total energy intake. These potentially confounding variables were measured at age 23–25 years.

In each adjusted regression model, possible interactions of the sex variable with the UPF consumption variable were evaluated, with those with a *p*-value of lower than 0.10 included in the final model. Based on the adjusted regression models, the risk of MetS and each of its components were estimated according to UPF consumption. Marginal graphs were generated to visualize these interactions. To investigate nonlinear effects, regression models were also considered with the UPF consumption variable in the quadratic form.

### 2.7. Ethical Aspects

The study was approved by the Research Ethics Committee of Hospital das Clínicas of the University of São Paulo’s School of Medicine of Ribeirão Preto (Note No. 1929/2000 CEP/SPC of 26 July 2000; Opinion No. 1.282.710 of 21 September 2015). All participants signed the informed consent form.

## 3. Results

Of the 896 participants evaluated in the main analyses, at 23–25 years, 55.7% were female, most were white and had 9 to 11 years of schooling. Regarding lifestyle, 89.8% reported drinking alcohol and 14.8% were smokers. Regarding food consumption, the mean UPF consumption in %kcal and %g was 38.3% and 35.6%, respectively. For the UPF consumption in %kcal, we observed a significant difference regarding sex and skin color, with higher UPF consumption among women and individuals of black skin color. Considering the UPF consumption in %g, we observed a significantly higher UPF consumption among individuals who did not consume alcohol and had a low physical activity level (Table 1).

The incidence of MetS at 37–39 years was 35.4%. Considering each of the MetS components alone, the incidences were: 63.0% of increased WC; 27.5% of elevated triglycerides; 35.9% of low HDL-c; 28.3% of increased blood pressure; and 29.4% of high glucose. The incidence of MetS and its components showed some significant differences regarding the demographic, socioeconomic, and lifestyle variables of participants at 23–25 years. Regarding gender, men showed significantly higher incidences of MetS, elevated triglycerides, increased blood pressure, and increased glucose, whereas women showed a higher incidence of low HDL-c. Regarding skin color, the incidence of increased WC was significantly higher among individuals of black skin color. Individuals with lower schooling, in turn, showed significantly higher incidences of MetS, increased WC, and increased glucose. Regarding marital status, individuals who had a partner showed a significantly higher incidence of increased WC. Participants with lower income showed significantly higher incidences of MetS, increased WC, and low HDL-c. In addition, we observed significantly higher incidences of increased triglycerides and low HDL-c among smokers and those who consumed alcoholic beverages, respectively (Table 2).

**Figure 1:**
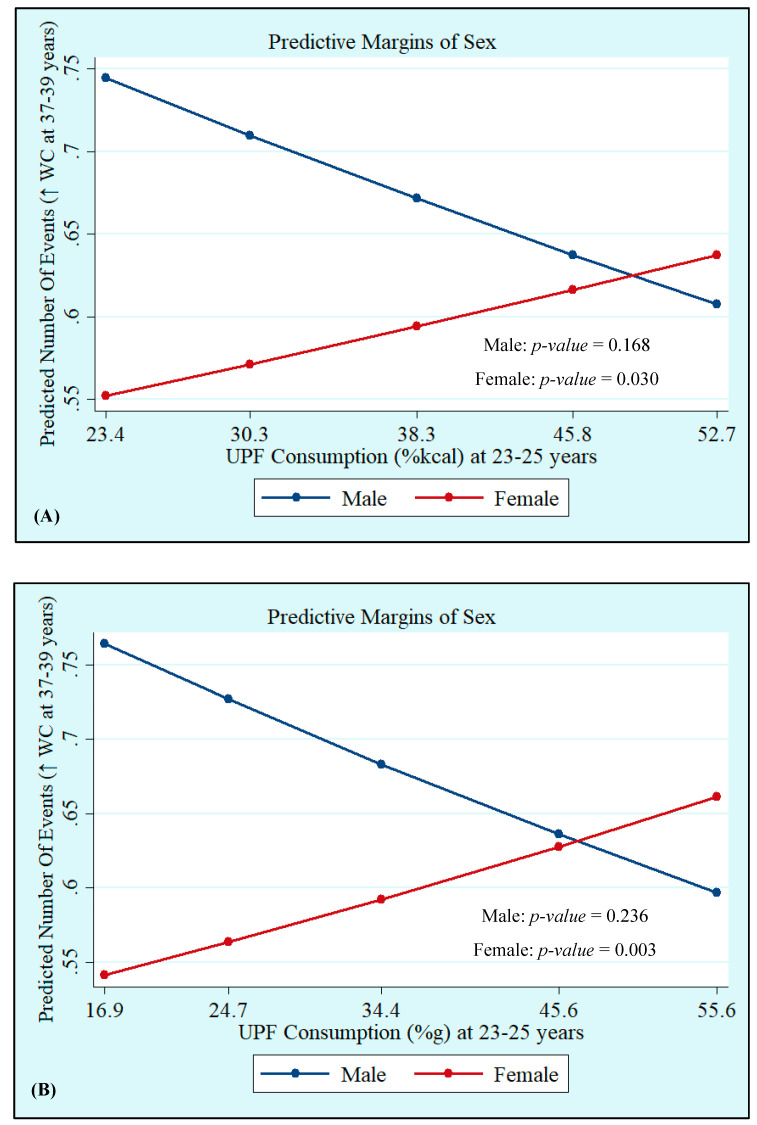
Incidence of high waist circumference (WC) at 37–39 years by sex according to ultra-processed food (UPF) consumption in %kcal (**A**) and %g (**B**) at 23–25 years. Ribeirão Preto Cohort 1978/79, São Paulo, Brazil.

**Figure 2:**
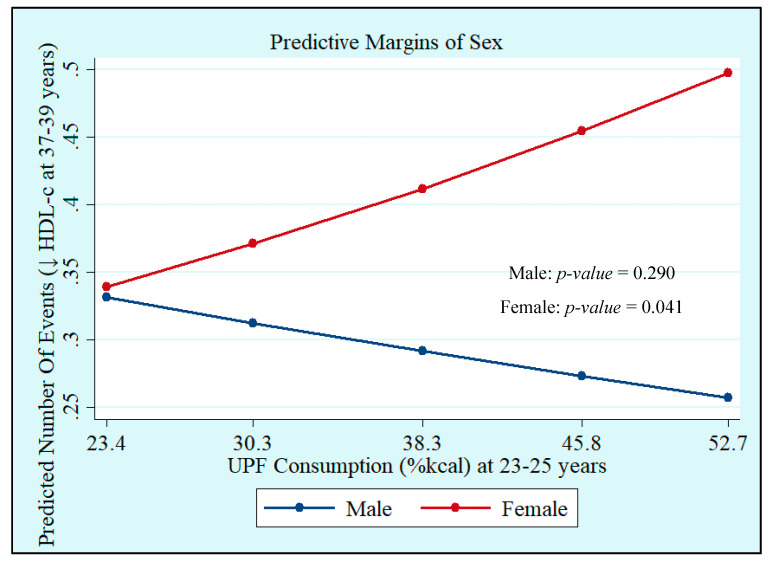
Incidence of low high-density lipoprotein-cholesterol (HDL-c) at 37–39 years by sex according to ultra-processed food (UPF) consumption at 23–25 years in %kcal. Ribeirão Preto Cohort 1978/79, São Paulo, Brazil.

Crude and adjusted analyses showed that UPF consumption at 23–25 years, both in %kcal and %g, was not associated with MetS at 37–39 years. However, we observed interactions with sex, which showed that UPF consumption in %kcal and %g was positively associated with increased WC incidence (%kcal: Male: *p* = 0.168 and Female: *p* = 0.030; %g: Male: *p* = 0.236 and Female: *p* = 0.003), whereas UPF consumption in %kcal was positively associated with low HDL-c (Male: *p* = 0.290 and Female: *p* = 0.041), with both associations being significant only in women (Table 3, Figure 1 and Figure 2). Quadratic terms were added to the models but were not statistically significant. Thus, they were not retained.

## 4. Discussion

This study found a mean caloric and in grams contribution of UPF consumption of 23–25 years of 38.3% and 35.6%, respectively. At 37–39 years of age, we observed high incidences of MetS (35.4%), abdominal obesity (62.5%), and low HDL-c (34.6%). The UPF consumption was not associated with MetS; however, the incidence of increased WC (in %kcal and %g) and low HDL-c (%kcal) was positively associated in women.

Regarding the caloric contribution of UPF consumption in adults, the literature is quite heterogeneous. In Brazil, surveys conducted in several regions of the country reported lower means of the caloric proportion of UPF consumption in adults compared to those observed in our study [28,29]. In the study conducted by Canella et al. [28], using data from the Household Budget Survey (2008–2009) obtained from a sample of individuals aged ≥ 10 years, the mean consumption of UPF corresponded to 20.5% of total calories. Furthermore, Simões et al. [29], in a study conducted with data from the Brazilian Longitudinal Study of Adult Health (ELSA-Brasil 2008–2010) with individuals aged between 35 and 74 years, showed that UPF consumption represented 22.7% of total caloric intake.

Substantially higher UPF consumption was observed in our study when compared to other studies carried out in Brazil, which may be related to the socioeconomic profile of the sample studied in the present study. Studies in Brazil already demonstrated that UPF consumption is associated with higher income and education [30,31]. Ribeirão Preto is one of the richest cities in the state of São Paulo and the participants of the 1978/79 Ribeirão Preto cohort have predominantly high income and high levels of education. On the other hand, the other studies cited provided a mean estimate of UPF consumption from different locations in the country, including places characterized by a population predominantly with lower income and education, which may have contributed to lower estimates of the consumption of these foods in these studies.

In the international scenario, Julia et al. [32] reported a mean close to that observed in our study, observing, when evaluating a sample of 74,470 French individuals aged ≥ 18 years in the NutriNet-Santé cohort, a mean UPF consumption of 35.9% of total energy intake. In contrast, other studies conducted with adult individuals in the UK [33], USA [34], and Canada [35] reported higher mean energy contribution of UPF consumption when compared with our study, ranging from 51% [35] to 59%. [34] In Korea, according to the Korea National Health and Nutrition Examination Survey (2010–2018), the mean energy contribution in the 20–49 age group was 27.7% [36].

Regarding the contribution in grams of UPF consumption, to our knowledge, so far only one Brazilian study reported such estimates in adults. Silva et al. [37], when evaluating a sample of 506 individuals aged 20 years or older living in the municipality of Brasília, the capital of Brazil, observed a mean contribution in grams of UPF consumption of 9.2%. In other countries, studies published so far evaluating UPF consumption in adults considering its proportion in grams were based on data from the NutriNet-Santé cohort study conducted in France [32,38,39,40,41,42,43], and reported percentages of contribution in grams ranging from 14.4% [38] to 18.7% [39]. Ratifying the arguments of such studies, when assessing UPF consumption considering its proportion in grams, it is important to consider UPFs that have lower or no energy value such as artificially sweetened beverages and non-nutritional factors related to food processing, such as neoformed contaminants, food additives, and changes in the structure of raw foods.

Regarding the incidence of MetS and its components, comparisons with some studies in the literature are limited due to the various criteria for defining MetS used among the authors and the fact that most studies estimated prevalence rather than incidence. Regarding the definition criteria of the MetS, note that when compared with the criteria of the National Cholesterol Education Program Adult Treatment Panel-Adult Treatment Panel III (NCEP-ATPIII) [44] and the American Heart Association/National Heart, Lung, and Blood Institute (AHA/NHLBI) [45], for most ethnic groups (including those in Central and South America), the criterion established by the Joint Interim Statement (JIS) [23] considers smaller cutoff points for the definition of high waist circumference. Regarding the criterion established by the International Diabetes Federation (IDF) [46], despite having the same cutoff points for the components, whereas the JIS [23] considers at least three altered parameters among any of the five components, the IDF includes abdominal obesity as a mandatory component alongside two other altered parameters to define MetS. These differences contribute to the identification of a greater number of individuals with high waist circumference and MetS with the criteria established by JIS, as indicated by several studies in the literature [47,48,49,50,51].

When comparing our findings with other studies that estimated incidences and considered the JIS criterion in the definition of MetS [52,53,54,55,56,57,58], very heterogeneous results were found, with MetS incidence ranging from 3.8% [56] to 49.8% [55]. When considering high WC and low HDL-c alone, the results were also discrepant compared with our findings [52,55,57], with incidences of abdominal obesity and low HDL-c ranging from 14.7% [52] to 47.2% [57] and from 24.5% [55] to 42.5% [52], respectively.

The discrepancies in the MetS, abdominal obesity, and low HDL-c estimates, reported among the studies that evaluated such outcomes considering the JIS criteria can be explained, in part, by the differences in the age groups of the participants and the follow-up time for measuring new cases in each study, which varied widely.

Regarding the association of UPF consumption and abdominal obesity in women, previous studies have reported similar findings; however, none of them evaluated the association longitudinally and estimated the incidences as our study did. In the study conducted by Sung et al. [59], with data from 7364 individuals aged 19–64 years participating in the Korea National Health and Nutrition Examination Survey (KNHANES), 2016–2018, only females showed a significant association, and women with higher UPF consumption (fourth quartile of %kcal of UPF) had a 64% higher abdominal obesity odds when compared with those with lower consumption (first quartile). In another study, with data from 15,977 American adults aged 20–64 participating in the National Health and Nutrition Examination Survey 2005–2014 [60], despite the significant association observed between UPF consumption and abdominal obesity in both sexes, a more pronounced association was reported among women. Regarding women in the lowest quintile of UPF consumption, women from the second to the fifth quintile had significantly higher odds of abdominal obesity. In contrast, in men, the associations were significant only for men in the highest quintile of UPF consumption compared with those in the first quintile.

The differences between the sexes in the associations observed are still unclear; however, the literature suggests that women are more predisposed to the adverse metabolic effects of foods rich in carbohydrates with a high glycemic index and glycemic load than men [61]. In addition, highlighting the differences in the type of UPF consumed between the sexes is important. In the sample excluding those who already had increased WC at 23–25 years of age in our study, in addition to a higher total UPF consumption, we observed higher proportions (in %kcal and %g) in the consumption of UFP subgroups that are rich in faster-digesting carbohydrates, such as breads, biscuits, cakes, sweets and desserts in women compared to men (Supplementary Table S1). Thus, a higher consumption of these types of UPF added to the greater sensitivity to the hyperglycemic effects of these foods in women, which could explain the effect of UPF consumption in the abdominal obesity observed only in females.

Previous studies investigated the association between UPF consumption (%kcal) and low HDL-c in adults; however, none of them evaluated the outcome by longitudinally estimating its incidences. UPF consumption was positively associated with low HDL-c in the studies conducted by Lavigne-Robichaud et al. [10] (Adjusted OR: 2.05; 95% CI: 1.25–3.38) in Canada, and by Martínez Steele et al. [9] (Adjusted RP: 1.34; 95% CI: 1.19–1.49) in the USA. On the other hand, Nasreddine et al. [62] did not observe a significant association between UPF consumption and low HDL-c in Lebanese adults (adjusted OR: 1.82; 95% CI: 0.52–6.42).

The association of UPF consumption (%kcal) and the higher incidence of low HDL-c observed only in women may be related to differences in food consumption between genders. According to the Update of the Brazilian guideline of dyslipidemia and prevention of atherosclerosis, 2017 [63], a diet rich in foods with a high content of saturated fats or *trans* fats tends to reduce HDL-c levels, with possible detriment to the functionality of HDL particles; foods that are carbohydrate sources with high glycemic index can also decrease HDL-c levels. In fact, a significantly higher UPF consumption (%kcal) in the subgroups of dairy products, margarines, breads, biscuits, cakes, sweets, and desserts was found in women compared to men in the sample excluding those with low HDL-c at 23–25 years in our study (Supplementary Table S2), which could contribute to the lowest levels of HDL-c in females.

Regarding the absence of a significant association of UPF consumption evaluated in %g and higher incidence of low HDL-c in females, we suggest that this may be related to the lower density (d = m/v) of some foods that may favor the reduction in HDL-c levels such as margarines, thereby impeding the analysis of UPF consumption considering the contribution in grams from capturing such an association.

Some limitations of this study must be considered. At the time, the NOVA classification had not yet been published, so the FFQ used to evaluate food consumption was not developed to classify food according to the degree of processing and, thus, the limitation of this instrument for measuring AUP consumption needs to be taken into account. In addition, food consumption was only considered at one point in time (because the FFQs used in each cohort follow-up were very different), thus it would not be surprising if intake modified over a lifetime. Another limitation concerns the evaluation of biochemical components at 37–39 years without fasting requirements during the blood sample collection. However, the literature shows that glucose, regardless of fasting in normoglycemic individuals, is usually <100 mg/dL [25]. Furthermore, for triglycerides, considering the high postprandial influence, we used the reference value for sample collection without fasting as a cutoff point instead of the parameter originally established by JIS [24,64]. Data were not adjusted for pharmacological treatment; however, the use of antilipemic, antihypertensive and antihyperglycemic drugs was considered to determine the MetS components, as described in the Methods. Regarding the presence of the same condition at the family level, we did not adjust for this because such data were not collected in our study.

On the other hand, our study has strengths. Its longitudinal character allowed us to evaluate the effect of UPF consumption on the incidence of MetS and its components over 15 years of follow-up of a sample of Brazilian adults. Another strength is the use of DAG in the construction of the theoretical model to define the variables used in the adjustment as confounders, thus reducing unnecessary adjustments.

## 5. Conclusions

In conclusion, our findings suggest no association between UPF consumption and MetS; however, the consumption of these foods was associated with two components of MetS, increased WC and low HDL-c only in women. The possible difference in dietary composition between men and women and its consequent impact on the development of cardiometabolic outcomes deserves further investigation. Thus, further studies on the topic are needed to elucidate this issue.

## Figures and Tables

**Table 1:** Mean and standard deviation (SD) of the ultra-processed food (UPF) consumption at 23–25 years according to demographic, socioeconomic, and lifestyle characteristics of the participants at 23–25 years. Ribeirão Preto Cohort 1978/79, São Paulo, Brazil.

		UPF Consumption (%kcal)		UPF Consumption (%g)	
Variables	N (%)	Mean (SD)	*p*-Value	Mean (SD)	*p*-Value
**Sex**			<0.0001 ^a^		0.2592 ^a^
Male	397 (44.3)	36.2 (10.8)		35.0 (14.1)	
Female	499 (55.7)	39.9 (11.1)		36.1 (15.8)	
**Skin color**			0.0108 ^b^		0.3339 ^b^
White	583 (65.1)	38.7 (11.1)		36.0 (15.3)	
Black	47 (5.3)	39.4 (10.6)		36.6 (14.2)	
Mixed Race	255 (28.5)	36.3 (11.8)		34.7 (15.2)	
Asian or Indigenous	11 (1.2)	40.9 (8.7)		41.7 (12.9)	
**Age (years)**			0.6337 ^b^		0.8402 ^b^
23	273 (30.5)	37.6 (11.8)		35.7 (15.9)	
24	447 (49.9)	38.3 (11.2)		35.5 (14.7)	
25	176 (19.6)	38.4 (11.0)		36.2 (15.2)	
**Education (years of study)**			0.1742 ^b^		0.1281 ^b^
0 to 8	111 (12.4)	37.5 (12.8)		37.9 (17.8)	
9 to 11	461 (51.5)	37.7 (11.6)		35.7 (15.2)	
≥12	324 (36.2)	39.0 (10.3)		34.8 (13.9)	
**Marital status**			0.6555 ^a^		0.1054 ^a^
With partner	269 (30.0)	38.0 (11.6)		36.9 (15.9)	
Without partner	627 (70.0)	38.4 (10.9)		35.1 (14.6)	
Family income (minimum wages) *			0.1687 ^b^		0.8880 ^b^
<5	287 (34.5)	37.1 (11.2)		35.3 (15.6)	
5 to 9.9	285 (34.3)	38.5 (11.6)		35.8 (14.7)	
≥10	259 (31.2)	38.9 (10.8)		35.3 (14.6)	
**Alcohol consumption**			0.0682 ^a^		0.0139 ^a^
No	91 (10.2)	40.3 (12.2)		39.3 (16.6)	
Yes	805 (89.8)	38.1 (11.0)		35.2 (14.8)	
**Smoking**			0.9006 ^a^		0.1077 ^a^
No	763 (85.2)	38.3 (11.0)		35.3 (14.8)	
Yes	133 (14.8)	38.3 (12.0)		37.6 (16.4)	
Physical activity level *			0.0507 ^b^		0.0004 ^b^
High	438 (49.0)	37.3 (11.3)		34.2 (14.6)	
Moderate	278 (31.1)	38.4 (11.3)		35.9 (15.7)	
Low	178 (19.9)	39.5 (11.2)		39.1 (15.1)	
**Total**	896 (100.0)	38.3 (11.1)		35.6 (15.0)	

^a^ Student’s *t*-test; ^b^ ANOVA. * Missing data.

**Table 2:** Incidence of metabolic syndrome and its components at 37–39 years according to demographic, socioeconomic and lifestyle characteristics of the participants at 23–25 years. Ribeirão Preto Cohort 1978/79, São Paulo, Brazil.

	MetS		↑ WC		↑ Triglycerides		↓ HDL-c		↑ BP		↑ Glucose	
Variables	N (%)	*p*-Value	N (%)	*p*-Value	N (%)	*p*-Value	N (%)	*p*-Value	N (%)	*p*-Value	N (%)	*p*-Value
**Sex**		<0.0001 ^a^		0.074 ^a^		<0.0001 ^a^		0.001 ^a^		<0.0001 ^a^		0.013 ^a^
Male	179 (45.1)		205 (66.1)		171 (41.7)		84 (28.4)		122 (39.7)		136 (30.0)	
Female	138 (27.7)		220 (59.5)		64 (13.2)		119 (41.0)		97 (19.2)		121 (23.1)	
**Skin color**		0.961 ^b^		0.003 ^b^		0.784 ^b^		0.334 ^b^		0.517 ^b^		0.705 ^b^
White	204 (35.0)		250 (58.3)		149 (25.9)		123 (32.6)		136 (25.5)		167 (25.9)	
Black	18 (38.3)		26 (76.5)		13 (28.3)		9 (29.0)		12 (28.6)		16 (33.3)	
Mixed race	91 (35.7)		145 (70.1)		72 (27.3)		68 (40.0)		3 (37.5)		71 (25.8)	
Asian or Indigenous	4 (36.4)		4 (40.0)		1 (11.1)		3 (37.5)				3 (27.3)	
**Age (years)**		0.845 ^a^		0.720 ^a^		0.561 ^a^		0.106 ^a^		0.898 ^a^		0.203
23	99 (36.3)		142 (64.3)		78 (28.7)		43 (27.7)		66 (26.2)		72 (24.3)	
24	154 (34.5)		200 (61.0)		112 (25.1)		117 (36.8)		110 (27.7)		123 (25.5)	
25	64 (36.4)		83 (63.4)		45 (25.4)		43 (38.1)		43 (26.4)		62 (31.2)	
**Education (years of study)**		0.002 ^a^		<0.0001 ^a^		0.084 ^a^		0.224 ^a^		0.109 ^a^		0.037 ^a^
0 to 8	45 (40.5)		50 (69.4)		32 (26.7)		25 (39.7)		31 (29.8)		26 (29.7)	
9 to 11	182 (39.5)		242 (69.3)		134 (29.1)		102 (37.0)		122 (29.3)		150 (20.8)	
≥12	90 (27.8)		133 (51.4)		69 (22.0)		76 (30.8)		66 (22.6)		81 (26.3)	
**Marital status**		0.559 ^a^		0.034 ^a^		0.199 ^a^		0.790 ^a^		0.693 ^a^		0.560 ^a^
With partner	99 (36.8)		123 (69.1)		65 (23.5)		52 (33.8)		70 (27.9)		82 (27.5)	
Without partner	218 (34.8)		302 (60.2)		170 (27.6)		151 (35.0)		149 (26.6)		175 (25.7)	
Family income (minimum wages) *		0.007 ^a^		<0.0001 ^a^		0.458 ^a^		0.037 ^a^		0.103 ^a^		0.055 ^a^
<5	119 (41.5)		144 (71.3)		84 (29.0)		71 (42.0)		83 (31.2)		90 (28.9)	
5 to 9.9	105 (36.8)		146 (65.8)		71 (24.4)		61 (35.5)		75 (28.4)		91 (28.7)	
≥10	74 (28.6)		104 (49.8)		67 (27.1)		61 (29.3)		50 (22.6)		59 (21.2)	
**Alcohol consumption**		0.852 ^a^		0.818 ^a^		0.132 ^a^		0.033 ^a^		0.592 ^a^		0.247 ^a^
No	33 (36.7)		44 (63.8)		17 (19.5)		25 (33.3)		25 (29.4)		20 (21.3)	
Yes	284 (35.3)		381 (62.4)		218 (27.0)		178 (48.1)		194 (26.7)		237 (26.8)	
**Smoking**		0.172 ^a^		0.737 ^a^		0.009 ^a^		0.237 ^a^		0.277 ^a^		0.949 ^a^
No	263 (34.7)		364 (62.8)		189 (24.7)		179 (35.6)		190 (27.7)		217 (26.2)	
Yes	54 (40.6)		61 (61.0)		46 (35.7)		24 (28.9)		29 (23.0)		40 (26.5)	
Physical activity level *		0.794 ^a^		0.164 ^a^		0.202 ^a^		0.863 ^a^		0.492 ^a^		0.222 ^a^
High	159 (36.3)		211 (61.9)		123 (28.0)		105 (35.4)		98 (26.1)		128 (26.9)	
Moderate	94 (33.8)		124 (59.1)		73 (26.7)		57 (33.0)		76 (29.6)		69 (22.9)	
Low	63 (35.4)		88 (69.3)		38 (21.1)		40 (35.1)		44 (24.9)		59 (29.7)	
**Total**	317/896 (35.4)		425/675 (63.0)		235/853 (27.5)		203/565 (35.9)		219/774 (28.2)		257/875 (29.4)	

MetS: Metabolic Syndrome; WC: Waist circumference; HDL-c: High-Density Lipoprotein-cholesterol; BP: Blood pressure. ^a^ Pearson’s chi-square test; ^b^ Fisher’s exact test. * Missing data.

**Table 3:** Crude and adjusted analysis of the association between ultra-processed food (UPF) consumption at 23–25 years and risk of metabolic syndrome and its components at 37–39 years. Ribeirão Preto Cohort 1978/79, São Paulo, Brazil.

Outcomes/Exposures/Interactions	Crude RR (95% CI)	Adjusted RR (95% CI)
**MetS**		
UPF (%kcal)	0.99 (0.98–1.00)	1.00 (0.99–1.01) ^a^
UPF (%g)	1.00 (0.99–1.01)	1.00 (0.99–1.01) ^b^
**↑ WC**		
UPF (%kcal)		
UPF	0.99 (0.98–1.00)	0.99 (0.98–1.00) ^a^
Sex	0.61 (0.40–0.93)	0.57 (0.38–0.85) ^a^
UPF##Sex	1.01 (1.00–1.02)	1.01 (1.00–1.02) ^a^
UPF (%g)		
UPF	1.00 (0.99–1.01)	0.99 (0.98–1.00) ^b^
Sex	0.60 (0.44–0.81)	0.57 (0.43–0.77) ^b^
UPF##Sex	1.01 (1.00–1.02)	1.01 (1.00–1.02) ^b^
**↑ Triglycerides**		
UPF (%kcal)	0.99 (0.98–1.00)	1.00 (0.99–1.01) ^a^
UPF (%g)	1.00 (0.99–1.01)	1.00 (0.99–1.01) ^b^
**↓ HDL-c**		
UPF (%kcal)		
UPF	0.99 (0.97–1.00)	0.99 (0.98–1.01) ^a^
Sex	0.61 (0.27–1.38)	0.66 (0.30–1.46) ^a^
UPF##Sex	1.02 (1.00–1.04)	1.02 (1.01–1.04) ^a^
UPF (%g)	0.99 (0.98–1.01)	0.99 (0.98–1.01) ^b^
**↑ BP**		
UPF (%kcal)	1.00 (0.99–1.01)	1.01 (1.00–1.02) ^a^
UPF (%g)	1.00 (0.99–1.01)	1.00 (0.99–1.01) ^b^
**↑ Glucose**		
UPF (%kcal)	1.00 (0.99–1.01)	1.00 (0.99–1.01) ^a^
UPF (%g)	1.00 (0.99–1.01)	0.99 (0.98–1.00) ^b^

MetS: Metabolic Syndrome; WC: Waist circumference; HDL-c: High-Density Lipoprotein-cholesterol; BP: Blood pressure; RR: Relative risk. ^a^ Adjusted for: Sex, skin color, age, education, marital status, family income, alcohol consumption, smoking, and level of physical activity. ^b^ Adjusted for: Sex, skin color, age, education, marital status, family income, alcohol consumption, smoking, level of physical activity and total caloric intake.

## Data Availability

The data presented in this study are available on request from the corresponding author.

## Supplementary Materials

The following are available online at https://www.mdpi.com/article/10.3390/nu14153126/s1, Figure S1: Directed Acyclic Graph (DAG) of the longitudinal association between ultraprocessed foods (UPF) consumption and metabolic syndrome (MetS), Table S1: Total ultraprocessed foods (UPF) consumption and its subgroups at 23–25 years according to sex in the sample excluding those who already had waist circumference increased (N = 680). Ribeirão Preto Cohort 1978/79, São Paulo, Brazil, Table S2: Total ultraprocessed foods (UPF) consumption and its subgroups at 23–25 years according to sex in the sample excluding those who already had low HDL-cholesterol (N = 586). Ribeirão Preto Cohort 1978/79, São Paulo, Brazil, Board S1: List of food items of Food Frequency Questionnaire (FFQ) applied to participants of 1978/79 Ribeirão Preto cohort in the follow-up at 23–25 years. Ribeirão Preto, São Paulo, Brazil.

## References

[1] Monteiro C.A., Cannon G., Lawrence M., Louzada M.L.d.C., Machado P.P. (2019). Ultra-Processed Foods, Diet Quality, and Health Using the NOVA Classification System.

[2] Organização Pan-Americana de Saúde (OPAS) (2018). Alimentos e Bebidas Ultraprocessados na América Latina: Tendências.

[3] Matos R.A., Adams M., Sabaté J. (2021). Review: The Consumption of Ultra-Processed Foods and Non-communicable Diseases in Latin America. Front. Nutr..

[4] Moore J.X., Chaudhary N., Akinyemiju T. (2017). Metabolic Syndrome Prevalence by Race/ Ethnicity and Sex in the United States, National Health and Nutrition Examination Survey, 1988–2012. Prev. Chronic. Dis..

[5] Wang H.H., Lee D.K., Liu M., Portincasa P., Wang D.Q.H. (2020). Novel insights into the pathogenesis and management of the metabolic syndrome. Pediatr. Gastroenterol. Hepatol. Nutr..

[6] Pérez-Martínez P., Mikhailidis D.P., Athyros V.G., Bullo M., Couture P., Covas M.I., de Koning L., Delgado-Lista J., Díaz-López A., Drevon C.A. (2017). Lifestyle recommendations for the prevention and management of metabolic syndrome: An international panel recommendation. Nutr. Rev..

[7] Lane M.M., Davis J.A., Beattie S., Gómez-Donoso C., Loughman A., O’Neil A., Jacka F., Berk M., Page R., Marx W. (2021). Ultraprocessed food and chronic noncommunicable diseases: A systematic review and meta-analysis of 43 observational studies. Obes. Rev..

[8] Pagliai G., Dinu M., Madarena M.P., Bonaccio M., Iacoviello L., Sofi F. (2021). Consumption of ultra-processed foods and health status: A systematic review and meta-Analysis. Br. J. Nutr..

[9] Martínez Steele E., Juul F., Neri D., Rauber F., Monteiro C.A. (2019). Dietary share of ultra-processed foods and metabolic syndrome in the US adult population. Prev. Med..

[10] Lavigne-Robichaud M., Moubarac J.C., Lantagne-Lopez S., Johnson-Down L., Batal M., Sidi E.A.L., Lucas M. (2018). Diet quality indices in relation to metabolic syndrome in an Indigenous Cree (Eeyouch) population in northern Québec, Canada. Public Health Nutr..

[11] Ivancovsky-Wajcman D., Fliss-Isakov N., Webb M., Bentov I., Shibolet O., Kariv R., Zelber-Sagi S. (2021). Ultra-processed food is associated with features of metabolic syndrome and non-alcoholic fatty liver disease. Liver Int..

[12] De Miranda R.C., Rauber F., Levy R.B. (2021). Impact of ultra-processed food consumption on metabolic health. Curr. Opin. Lipidol..

[13] Barbieri M.A., Ferraro A.A., Simões V.M.F., Goldani M.Z., Cardoso V.C., Moura da Silva A.A., Bettiol H. (2022). Cohort Profile: The 1978–79 Ribeirao Preto (Brazil) birth cohort study. Int. J. Epidemiol..

[14] Barbieri M.A., Bettiol H., Silva A.A.M., Cardoso V.C., Simões V.M.F., Gutierrez M.R.P., Castro J.A.S., Vianna E.S.O., Foss M.C., Dos Santos J.E. (2006). Health in early adulthood: The contribution of the 1978/79 Ribeirao Preto birth cohort. Brazilian J. Med. Biol. Res..

[15] Ribeiro A.B., Cardoso M.A. (2002). Development of a food frequency questionnaire as a tool for programs of chronic diseases prevention. Rev. Nutr..

[16] Monteiro J.P., Pfrimer K., Tremeschin M.H., Molina M.C., Chiarello P. (2007). Consumo Alimentar: Visualizando Porções.

[17] Zabotto C.B., Viana R.P.T., Gil M.F. Registro Fotográfico Para Inquéritos Dietéticos: Utensílios e Porções. https://www.fcm.unicamp.br/fcm/sites/default/files/2016/page/manual_fotografico.pdf.

[18] U.S. (2021). Department of Agriculture Food Data Central. https://www.nal.usda.gov/fnic/food-composition.

[19] Instituto Brasileiro de Geografia e Estatística—IBGE (2011). Pesquisa de Orçamentos Familiares 2008–2009: Tabela de Composição Nutricional dos Alimentos Consumidos No Brasil.

[20] Núcleo de Estudos e Pesquisas em Alimentação—NEPA (2011). Tabela Brasileira de Composição de Alimentos.

[21] Louzada M.L.d.C., Canella D.S., Jaime P.C., Monteiro C.A. (2019). Alimentação e Saúde: A Fundamentação Científica do Guia Alimentar para a População Brasileira.

[22] Instituto Brasileiro de Geografia e Estatística—IBGE (2004). Pesquisa de Orçamentos Familiares 2002–2003: Primeiros Resultados Brasil e Grandes Regiões.

[23] Alberti K.G.M.M., Eckel R., Grundy S., Zimmet P., Cleeman J., Donato K., Fruchart J.-C., James W.P.T., Loria C.M., Smith S.C. (2009). Harmonizing the metabolic syndrome: A joint interim statement of the International Diabetes Federation Task Force on Epidemiology and Prevention; National Heart, Lung, and Blood Institute; American Heart Association; World Heart Federation; International Atherosclerosis Society; and International Association for the Study of Obesity. Circulation.

[24] Nordestgaard B.G., Langsted A., Mora S., Kolovou G., Baum H., Bruckert E., Watts G.F., Sypniewska G., Wiklund O., Borén J. (2016). Fasting is not routinely required for determination of a lipid profile: Clinical and laboratory implications including flagging at desirable concentration cut-points—A joint consensus statement from the European Atherosclerosis Society and European Fede. Eur. Heart J..

[25] Bowen M.E., Xuan L., Lingvay I., Halm E.A. (2018). Doc, I Just Ate: Interpreting Random Blood Glucose Values in Patients with Unknown Glycemic Status. J. Gen. Intern. Med..

[26] IPAQ Committee (2005). Guidelines for Data Processing and Analysis of the International Physical Activity Questionnaire (IPAQ)—Short and Long Forms.

[27] Textor J., Hardt J., Knüppel S. (2011). DAGitty: A graphical tool for analyzing causal diagrams. Epidemiology.

[28] Canella D.S., Louzada M.L.d.C., Claro R.M., Costa J.C., Bandoni D.H., Levy R.B., Martins A.P.B. (2018). Consumption of vegetables and their relation with ultra-processed foods in Brazil. Rev. Saude Publica.

[29] Simões B.d.S., Barreto M.S., Molina M.d.C.B., Luft V.C., Ducan B.B., Schmidt M.I., Benseñor I.J.M., Cardoso L.d.O., Levy R.B., Giatti L. (2018). Consumption of ultra-processed foods and socioeconomic position: A cross-sectional analysis of the Brazilian Longitudinal Study of Adult Health (ELSA-Brasil). Cad. Saude Publica.

[30] Monteiro C.A., Levy R.B., Claro R.M., De Castro I.R.R., Cannon G. (2010). Increasing consumption of ultra-processed foods and likely impact on human health: Evidence from Brazil. Public Health Nutr..

[31] Bielemann R.M., Santos Motta J.V., Minten G.C., Horta B.L., Gigante D.P. (2015). Consumption of ultra-processed foods and their impact on the diet of young adults. Rev. Saude Publica.

[32] Julia C., Martinez L., Allès B., Touvier M., Hercberg S., Méjean C., Kesse-Guyot E. (2018). Contribution of ultra-processed foods in the diet of adults from the French NutriNet-Santé study. Public Health Nutr..

[33] Rauber F., Louzada M.L.D.C., Martinez Steele E., De Rezende L.F.M., Millett C., Monteiro C.A., Levy R.B. (2019). Ultra-processed foods and excessive free sugar intake in the UK: A nationally representative cross-sectional study. BMJ Open.

[34] Baraldi L.G., Martinez Steele E., Canella D.S., Monteiro C.A. (2018). Consumption of ultra-processed foods and associated sociodemographic factors in the USA between 2007 and 2012: Evidence from a nationally representative cross-sectional study. BMJ Open.

[35] Moubarac J.-C., Batal M., Louzada M.L., Steele E.M., Monteiro C.A. (2017). Consumption of ultra-processed foods predicts diet quality in Canada. Appetite.

[36] Shim J.S., Shim S.Y., Cha H.J., Kim J., Kim H.C. (2021). Socioeconomic characteristics and trends in the consumption of ultra-processed foods in Korea from 2010 to 2018. Nutrients.

[37] Silva C.L., Sousa A.G., Borges L.P.S.L., da Costa T.H.M. (2021). Usual consumption of ultra-processed foods and its association with sex, age, physical activity, and body mass index in adults living in Brasília city, Brazil. Rev. Bras. Epidemiol..

[38] Schnabel L., Kesse-Guyot E., Allès B., Touvier M., Srour B., Hercberg S., Buscail C., Julia C. (2019). Association between Ultraprocessed Food Consumption and Risk of Mortality among Middle-aged Adults in France. JAMA Intern. Med..

[39] Fiolet T., Srour B., Sellem L., Kesse-Guyot E., Allès B., Méjean C., Deschasaux M., Fassier P., Latino-Martel P., Beslay M. (2018). Consumption of ultra-processed foods and cancer risk: Results from NutriNet-Santé prospective cohort. Br. Med. J..

[40] Schnabel L., Buscail C., Sabate J.M., Bouchoucha M., Kesse-Guyot E., Allès B., Touvier M., Monteiro C.A., Hercberg S., Benamouzig R. (2018). Association Between Ultra-Processed Food Consumption and Functional Gastrointestinal Disorders: Results From the French NutriNet-Santé Cohort. Am. J. Gastroenterol..

[41] Srour B., Fezeu L.K., Kesse-Guyot E., Allès B., Méjean C., Andrianasolo R.M., Chazelas E., Deschasaux M., Hercberg S., Galan P. (2019). Ultra-processed food intake and risk of cardiovascular disease: Prospective cohort study (NutriNet-Santé). Br. Med. J..

[42] Srour B., Fezeu L.K., Kesse-Guyot E., Allès B., Debras C., Druesne-Pecollo N., Chazelas E., Deschasaux M., Hercberg S., Galan P. (2019). Ultraprocessed Food Consumption and Risk of Type 2 Diabetes among Participants of the NutriNet-Santé Prospective Cohort. JAMA Intern. Med..

[43] Adjibade M., Julia C., Allès B., Touvier M., Lemogne C., Srour B., Hercberg S., Galan P., Assmann K.E., Kesse-Guyot E. (2019). Prospective association between ultra-processed food consumption and incident depressive symptoms in the French NutriNet-Santé cohort. BMC Med..

[44] Expert Panel on Detection E. (2001). Executive summary of the Third Report of the National Cholesterol Education Programme. JAMA.

[45] Grundy S.M., Cleeman J.I., Daniels S.R., Donato K.A., Eckel R.H., Franklin B.A., Gordon D.J., Krauss R.M., Savage P.J., Smith S.C. (2005). Diagnosis and management of the metabolic syndrome: An American Heart Association/National Heart, Lung, and Blood Institute scientific statement. Circulation.

[46] Alberti K.G.M.M., Zimmet P., Shaw J. (2006). Metabolic syndrome—A new world-wide definition. A consensus statement from the International Diabetes Federation. Diabet. Med..

[47] Haverinen E., Paalanen L., Palmieri L., Padron-Monedero A., Noguer-Zambrano I., Suárez R.S., Tolonen H. (2021). Comparison of metabolic syndrome prevalence using four definitions—A case study from Finland. Eur. J. Public Health.

[48] do Vale Moreira N.C., Hussain A., Bhowmik B., Mdala I., Siddiquee T., Fernandes V.O., Montenegro Júnior R.M., Meyer H.E. (2020). Prevalence of Metabolic Syndrome by different definitions, and its association with type 2 diabetes, pre-diabetes, and cardiovascular disease risk in Brazil. Diabetes Metab. Syndr. Clin. Res. Rev..

[49] Esmailzadehha N., Ziaee A., Kazemifar A.M., Ghorbani A., Oveisi S. (2013). Prevalence of metabolic syndrome in Qazvin Metabolic Diseases Study (QMDS), Iran: A comparative analysis of six definitions. Endocr. Regul..

[50] Ramli A.S., Daher A.M., Nor-Ashikin M.N.K., Mat-Nasir N., Ng K.K., Miskan M., Ambigga K.S., Ariffin F., Mazapuspavina M.Y., Abdul-Razak S. (2013). JIS definition identified more malaysian adults with metabolic syndrome compared to the NCEP-ATP III and IDF criteria. Biomed. Res. Int..

[51] Adejumo E.N., Ogundahunsi O.A., Adejumo O.A., Sotunsa J., Jagun O. (2017). Prevalence of Metabolic Syndrome in a Rural and Urban Community in South-West Nigeria Using Three Different Definitions. Int. J. Trop. Dis. Health.

[52] Mirmiran P., Moslehi N., Hosseinpanah F., Sarbazi N., Azizi F. (2020). Dietary determinants of unhealthy metabolic phenotype in normal weight and overweight/obese adults: Results of a prospective study. Int. J. Food Sci. Nutr..

[53] Cheraghi Z., Nedjat S., Mirmiran P., Moslehi N., Mansournia N., Etminan M., Mansournia M.A., McCandless L.C. (2018). Effects of food items and related nutrients on metabolic syndrome using Bayesian multilevel modelling using the Tehran Lipid and Glucose Study (TLGS): A cohort study. BMJ Open.

[54] Hosseinpour-Niazi S., Hosseini S., Mirmiran P., Azizi F. (2017). Prospective study of nut consumption and incidence of metabolic syndrome: Tehran Lipid and glucose study. Nutrients.

[55] Babio N., Becerra-Tomás N., Martínez-González M.Á., Corella D., Estruch R., Ros E., Sayón-Orea C., Fitá M., Serra-Majem L., Arós F. (2015). Consumption of Yogurt, Low-FatMilk, and Other Low-Fat Dairy Products Is Associated with Lower Risk of Metabolic Syndrome Incidence in an Elderly Mediterranean Population1-3. J. Nutr..

[56] Sayón-Orea C., Bes-Rastrollo M., Martí A., Pimenta A.M., Martín-Calvo N., Martínez-González M.A. (2015). Association between yogurt consumption and the risk of Metabolic Syndrome over 6 years in the SUN study Disease epidemiology—Chronic. BMC Public Health.

[57] Yoon H.-J., Lee S.-K. (2015). The Incidence and Risk Factors of Metabolic Syndrome in Rural Area. J. Korea Acad. Coop. Soc..

[58] Mosley M.A., Andrade F.C.D., Aradillas-Garcia C., Teran-Garcia M. (2013). Consumption of Dairy and Metabolic Syndrome Risk in a Convenient Sample of Mexican College Applicants. Food Nutr. Sci..

[59] Sung H., Park J.M., Oh S.U., Ha K., Joung H. (2021). Consumption of ultra-processed foods increases the likelihood of having obesity in Korean women. Nutrients.

[60] Juul F., Martinez-Steele E., Parekh N., Monteiro C.A., Chang V.W. (2018). Ultra-processed food consumption and excess weight among US adults. Br. J. Nutr..

[61] Mirrahimi A., Chiavaroli L., Srichaikul K., Augustin L.S.A., Sievenpiper J.L., Kendall C.W.C., Jenkins D.J.A. (2014). The role of glycemic index and glycemic load in cardiovascular disease and its risk factors: A review of the recent literature. Curr. Atheroscler. Rep..

[62] Nasreddine L., Tamim H., Itani L., Nasrallah M.P., Isma’eel H., Nakhoul N.F., Abou-Rizk J., Naja F. (2018). A minimally processed dietary pattern is associated with lower odds of metabolic syndrome among Lebanese adults. Public Health Nutr..

[63] Faludi A.A., Izar M.C.O., Saraiva J.F.K., Chacra A.P.M., Bianco H.T., Afiune Neto A., Bertolami A., Pereira A.C., Lottenberg A.M., Sposito A.C. (2017). Diagnosis and treatment of rare complication after endomyocardial biopsy. Arq. Bras. Cardiol..

[64] Langsted A., Nordestgaard B.G. (2019). Nonfasting versus fasting lipid profile for cardiovascular risk prediction. Pathology.

