# Supplementary material for: Sex-Dependent Effects of the Intake of NOVA Classified Ultra-Processed Foods on Syndrome Metabolic Components in Brazilian Adults
Source: Nutrients. 2022 Jul 29;14(15):3126. doi: 10.3390/nu14153126 (PMC9370159; doi:10.3390/nu14153126)
Supplement: Supplementary file 1 [file nutrients-14-03126-s001.zip › nutrients-1803414-supplementary.pdf]

Board S1. List of food items of Food Frequency Questionnaire (FFQ) applied to participants of 1978/79 Ribeirão Preto cohort in the follow-up at 23-25 years. Ribeirão Preto, São Paulo, Brazil.

Whole milk
Skimmed milk
Semi-skimmed milk
Whole natural yogurt
Skimmed natural yogurt
Yogurt with fruits flavor
White cheese or ricotta
Yellow cheeses
Cream curd cheese
French bread, loaf bread
Wholemeal bread, rye bread
Sweet bread, cheese bread others
Sweet/savory biscuits or toasts
Oatmeal, granola and others
Regular margarine
Light margarine
Butter
Mayonnaise
White rice
Potatoes, cassava and polenta (fries)
Potatoes, cassava and polenta (other types of preparation)
Green corn
Sweet potato
Pasta (noodles, gnocchi)
Savory and pie (pizza)
Farofa[a], corn flour
Orange, tangerine
Banana
Apple, pear
Papaya
Watermelon, melon
Grape, pineapple, guava (in season)
Avocado (in season)
Mango, persimmon (in season)
Other fruits
Natural orange juice
Juice from other fruits

Purple beans, carioca beans
Peas, lentils, others
Feijoada [b]
Lettuce, endive, watercress, arugula, chicory
Cabbage, chard, kale, spinach
Broccoli, Cauliflower
Carrot, pumpkin
Tomato
Eggplant
Beetroot, pod, chayote, zucchini
Soups
Beef without fat
Beef with fat
Pork without fat
Pork with fat
Bacon, lard, crackling
Chicken, chester, turkey, other poultry (without skin)
Chicken, chester, turkey, other poultry (with skin)
Fishes
Offals of ox, poultry or pork, tripe
Shrimp, seafood
Pepperoni sausage, franks
Ham, bologna, others
Boiled egg
Fried egg
Brewed coffee without sugar/without sweetener
Brewed coffee with sugar
Brewed coffee with sweetener
Black or matt tea
Herbs tea
Water
Beer
Sugar cane brandy, distilled drinks
Wine
Artificial juices
Diet/light soft drink
Regular soft drink
Phosphated soft drink
Sweet cakes and pies
Chocolate, Brazilian sweet truffle
Honey, jam

Ice cream, milkshake
Pudding, sweets with milk
Sweets of fruits
Nuts and oilseeds
Popcorn, chips, others

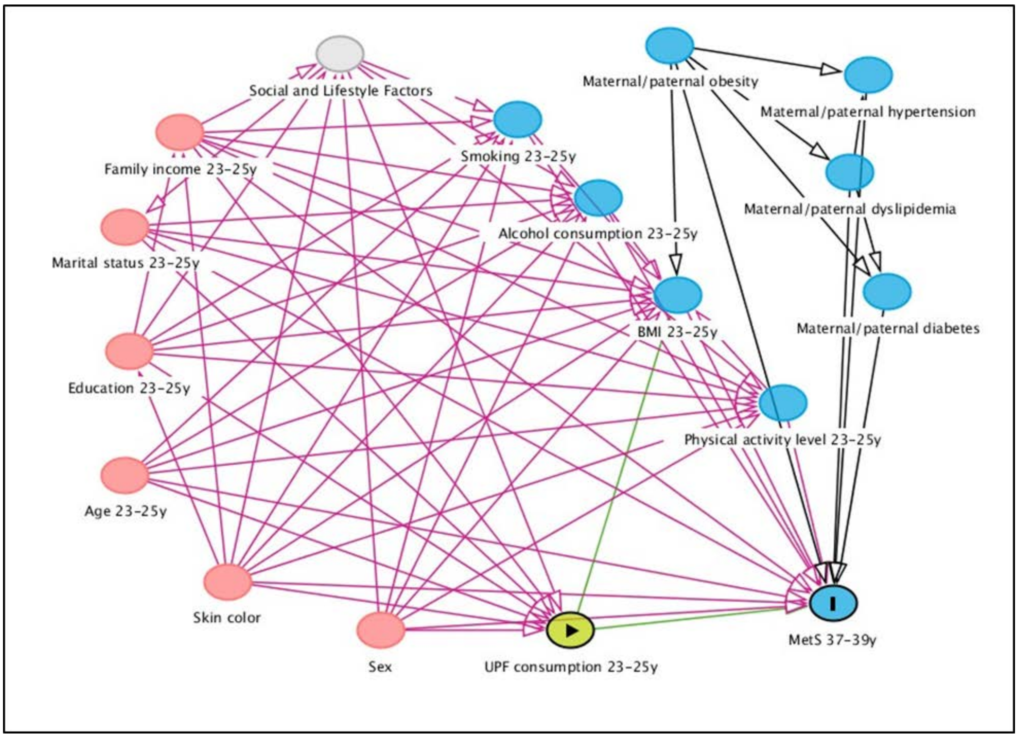

Figure S1. Directed Acyclic Graph (DAG) of the longitudinal association between ultraprocessed foods (UPF) consumption and metabolic syndrome (MetS).

Table S1. Total ultraprocessed foods (UPF) consumption and its subgroups at 23-25 years according to sex in the sample excluding those who already had waist circumference increased (N = 680). Ribeirão Preto Cohort 1978/79, São Paulo, Brazil.

UPF	%kcal			%g		
	Male	Female	*P*-value	Male	Female	*P*-value [a]
	Mean (SD)	Mean (SD)		Mean (SD)	Mean (SD)	
**UPF total**	36.2 (10.9)	40.0 (11.2)	**< 0.0001**	35.3 (14.2)	36.0 (15.5)	0.5186
**Dairy products** [b]	1.2 (1.6)	2.1 (2.5)	**< 0.0001**	1.0 (1.4)	1.9 (2.4)	**< 0.0001**
**Ultra-processed breads** [c]	1.8 (1.6)	2.4 (2.2)	**0.0001**	0.7 (0.7)	0.9 (0.8)	**0.0019**
**Biscuits** [d]	2.6 (2.7)	3.6 (3.8)	**0.0001**	0.6 (0.7)	0.9 (1.0)	**0.0002**
**Granola**	0.4 (1.1)	0.8 (1.7)	**0.0020**	0.1 (0.3)	0.2 (0.5)	0.0045
**Instant noodles**	1.0 (0.8)	1.1 (0.8)	0.1727	0.4 (0.4)	0.5 (0.4)	0.3290
**Margarines** [e]	0.8 (1.0)	0.9 (1.2)	0.2701	0.1 (0.2)	0.2 (0.2)	0.0976
**Mayonnaise**	0.5 (0.7)	0.4 (0.7)	0.1174	0.2 (0.2)	0.2 (0.3)	0.2155
**Salty snacks** [f]	9.2 (7.3)	7.3 (6.3)	**0.0003**	4.1 (3.7)	3.1 (3.0)	**0.0003**
**Sausage meats** [g]	1.9 (1.8)	1.6 (1.7)	**0.0232**	1.2 (1.0)	1.0 (0.9)	**0.0225**
**Soft drinks and artificial juices**	9.7 (6.6)	10.3 (8.6)	0.2883	24.4 (13.6)	24.2 (15.7)	0.8447
**Distilled beverages** [h]	0.1 (0.4)	0.1 (0.3)	**0.0364**	0.1 (0.2)	0.04 (0.1)	**0.0304**
**Sweet cakes and pies**	1.7 (2.1)	2.1 (2.7)	**0.0104**	0.7 (0.9)	0.9 (1.3)	**0.0248**
**Sweets and desserts** [i]	3.5 (3.5)	5.0 (4.2)	**< 0.0001**	1.1 (1.2)	1.5 (1.3)	**0.0002**
**Chips**	1.6 (2.8)	1.9 (2.7)	0.1270	0.4 (1.0)	0.4 (0.7)	0.3372

[a] Student’s t-test.

[b] Yogurt sweetened or artificially flavored and industrialized cream curd cheese; [c] Industrialized breads: wholemeal bread, white bread and sweet bread; [d] Industrialized sweet and savory biscuits; [e] Regular and light margarine; [f] Pizza and savory pies; [g] Pepperoni sausage, franks, ham and bologna; [h] Sugar cane brandy, vodka and whiskey; [i] Chocolate, Brazilian sweet truffle, ice cream, milkshake, pudding, sweets with milk.

Table S2. Total ultraprocessed foods (UPF) consumption and its subgroups at 23-25 years according to sex in the sample excluding those who already had low HDL-cholesterol (N = 586). Ribeirão Preto Cohort 1978/79, São Paulo, Brazil.

UPF	%kcal		*P*-value [a]
	Male	Female	
	Mean (SD)	Mean (SD)	
**UPF total**	35.5 (10.5)	39.9 (11.1)	**< 0.0001**
**Dairy products** [b]	1.1 (1.4)	2.0 (2.3)	**< 0.0001**
**Ultra-processed breads** [c]	1.9 (1.7)	2.5 (2.2)	**0.0009**
**Biscuits** [d]	2.4 (2.6)	3.7 (4.0)	**< 0.0001**
**Granola**	0.5 (1.3)	0.7 (1.7)	0.1226
**Instant noodles**	1.0 (0.8)	1.1 (0.9)	0.0695
**Margarines** [e]	0.7 (0.9)	0.9 (1.1)	**0.0285**
**Mayonnaise**	0.5 (0.7)	0.5 (0.8)	0.3143
**Salty snacks** [f]	9.1 (6.9)	7.2 (6.4)	**0.0006**
**Sausage meats** [g]	2.0 (1.8)	1.6 (1.6)	**0.0124**
**Soft drinks and artificial juices**	9.4 (6.2)	10.1 (8.8)	0.2404
**Distilled beverages** [h]	0.2 (0.4)	0.1 (0.3)	**0.0017**
**Sweet cakes and pies**	1.5 (1.8)	2.2 (2.6)	**0.0006**
**Sweets and desserts** [i]	3.8 (3.8)	5.3 (4.4)	**< 0.0001**
**Chips**	1.5 (2.7)	1.9 (2.8)	0.0512

[a] Student's t-test.

[b] Yogurt sweetened or artificially flavored and industrialized cream curd cheese; [c] Industrialized breads: wholemeal bread, white bread and sweet bread; [d] Industrialized sweet and savory biscuits; [e] Regular and light margarine; [f] Pizza and savory pies; [g] Pepperoni sausage, franks and ham; [h] Sugar cane brandy, vodka and whiskey; [i] Chocolate, ice cream, milkshake, pudding, sweets with milk.
